# Supplementary material for: Adaptive dating and fast proposals: Revisiting the phylogenetic relaxed clock model
Source: PLoS Comput Biol. 2021 Feb 2;17(2):e1008322. doi: 10.1371/journal.pcbi.1008322 (PMC7880504; doi:10.1371/journal.pcbi.1008322)
Supplement: S1 Appendix — The linear piecewise approximation used in the quant parameterisation is described. Constant distance tree operators, CisScale, and NarrowExchangeRate are extended to the quant parameterisation. A second NER algorithm is specified. (PDF) [file pcbi.1008322.s001.pdf]

Adaptive dating and fast proposals: revisiting the phylogenetic relaxed clock model

Jordan Douglas[1,2*], Rong Zhang[1,2], Remco Bouckaert[1,2,3]

1 Centre for Computational Evolution, University of Auckland, Auckland, New Zealand
2 School of Computer Science, University of Auckland, Auckland, New Zealand
3 Max Planck Institute for the Science of Human History, Jena, Germany

* jordan.douglas@auckland.ac.nz

S1 Appendix: Rate quantiles and operators

1 Piecewise linear approximation

In this article we introduced a linear piecewise approximation of the i-CDF (inverse cumulative distribution function) to improve the computational performance of the *quant* parameterisation. Let $\hat{F}^{-1}(\mathcal{R}_i)$ be the piecewise approximation of the i-CDF $F^{-1}(\mathcal{R}_i)$. The approximation consists of n pieces (where $n = 100$ is fixed). Due to the nonlinear nature of small and large quantiles in a log-normal distribution, the first and last pieces are not linear approximations but rather equal to the underlying distribution itself.

$$\hat{F}^{-1}(q) = \begin{cases} F^{-1}(q) & \text{if } q \leq \frac{1}{n} \text{ or } q \geq \frac{n-1}{n} \\ F^{-1}(\lfloor v \rfloor) + \Big(F^{-1}(\lfloor v \rfloor + 1) - F^{-1}(\lfloor v \rfloor)\Big)\Big(v - \lfloor v \rfloor\Big) & \text{otherwise.} \end{cases} \quad (1)$$

where $v = q(n-1)$ indexes quantile q into piece number $\lfloor v \rfloor$. Values from the underlying function F^{-1} are cached, enabling rapid computation.

2 Tree operators for rate quantiles

Zhang and Drummond 2020 introduced several tree operators for the *real* parameterisation – including `ConstantDistance`, `SimpleDistance`, and `SmallPulley` [1]. In this appendix, these three operators are extended to the *quant* parameterisation. Following the notation presented in the main article, let t_i be the time of node i, let $0 < q_i < 1$ be the rate quantile of node i, and let $r_i = \hat{F^{-1}}(q_i)$ be the real rate of node i where $\hat{F^{-1}}$ is the linear approximation of the i-CDF.

Constant Distance

Let $\mathcal{X}$ be a uniformly-at-random sampled internal node on tree $\mathcal{T}$. Let $\mathcal{L}$ and $\mathcal{R}$ be the left and right child of $\mathcal{X}$, respectively, and let $\mathcal{P}$ be the parent of $\mathcal{X}$. Under the *quant* parameterisation, the `ConstantDistance` operator works as follows:

Step 1. Propose a new height for $t_{\mathcal{X}}$:

$$t_{\mathcal{X}}' \leftarrow t_{\mathcal{X}} + s\Sigma \tag{2}$$

where Σ is drawn from a proposal transition distribution (Uniform or Bactrian), and s is a tunable step size. Ensure that $\max\{t_{\mathcal{L}}, t_{\mathcal{R}}\} < t_{\mathcal{X}}' < t_{\mathcal{P}}$, and if the constraint is broken then reject the proposal.

Step 2. Recalculate $q_{\mathcal{X}}$ as:

$$\begin{aligned} q_{\mathcal{X}}' \leftarrow & \hat{F}\Big(r_{\mathcal{X}}'\Big) \\ \leftarrow & \hat{F}\Big(\frac{t_{\mathcal{P}} - t_{\mathcal{X}}}{t_{\mathcal{P}} - t_{\mathcal{X}}'} r_X\Big) \\ \leftarrow & \hat{F}\Big(\frac{t_{\mathcal{P}} - t_{\mathcal{X}}}{t_{\mathcal{P}} - t_{\mathcal{X}}'} \hat{F^{-1}}(q_{\mathcal{X}})\Big). \end{aligned} \tag{3}$$

This ensures that the genetic distance between $\mathcal{X}$ and P remains constant after the operation by enforcing the constraint:

$$r_{\mathcal{X}}(t_{\mathcal{P}} - t_{\mathcal{X}}) = r_{\mathcal{X}}'(t_{\mathcal{P}} - t_{\mathcal{X}}'). \tag{4}$$

Step 3. Similarly, propose new rate quantiles for the two children $\mathcal{C} \in \{\mathcal{L}, \mathcal{R}\}$:

$$\begin{aligned} q_{\mathcal{C}}' \leftarrow & \hat{F}\Big(r_{\mathcal{C}}'\Big) \\ \leftarrow & \hat{F}\Big(\frac{t_{\mathcal{X}} - t_{\mathcal{C}}}{t_{\mathcal{X}}' - t_{\mathcal{C}}} \times r_{\mathcal{C}}\Big) \\ \leftarrow & \hat{F}\Big(\frac{t_{\mathcal{X}} - t_{\mathcal{C}}}{t_{\mathcal{X}}' - t_{\mathcal{C}}} \times \hat{F^{-1}}(q_{\mathcal{C}})\Big). \end{aligned} \tag{5}$$

Ensure that $0 < q_i' < 1$ for all proposed nodes $i \in \{\mathcal{X}, L, R\}$, and if the constraint is broken then reject the proposal. This constraint can only be broken from numerical issues.

Step 4. Finally, in order to calculate the Metropolis-Hastings-Green ratio, return the determinant of the Jacobian matrix:

$$J = \begin{bmatrix} \frac{\partial t_{\mathcal{X}}'}{\partial t_{\mathcal{X}}} & \frac{\partial t_{\mathcal{X}}'}{\partial q_{\mathcal{X}}} & \frac{\partial t_{\mathcal{X}}'}{\partial q_{\mathcal{L}}} & \frac{\partial t_{\mathcal{X}}'}{\partial q_{\mathcal{R}}} \\ \frac{\partial q_{\mathcal{X}}'}{\partial t_{\mathcal{X}}} & \frac{\partial q_{\mathcal{X}}'}{\partial q_{\mathcal{X}}} & \frac{\partial q_{\mathcal{X}}'}{\partial q_{\mathcal{L}}} & \frac{\partial q_{\mathcal{X}}'}{\partial q_{\mathcal{R}}} \\ \frac{\partial q_{\mathcal{L}}'}{\partial t_{\mathcal{X}}} & \frac{\partial q_{\mathcal{L}}'}{\partial q_{\mathcal{X}}} & \frac{\partial q_{\mathcal{L}}'}{\partial q_{\mathcal{L}}} & \frac{\partial q_{\mathcal{L}}'}{\partial q_{\mathcal{R}}} \\ \frac{\partial q_{\mathcal{R}}'}{\partial t_{\mathcal{X}}} & \frac{\partial q_{\mathcal{R}}'}{\partial q_{\mathcal{X}}} & \frac{\partial q_{\mathcal{R}}'}{\partial q_{\mathcal{L}}} & \frac{\partial q_{\mathcal{R}}'}{\partial q_{\mathcal{R}}} \end{bmatrix} = \begin{bmatrix} \frac{\partial t_{\mathcal{X}}'}{\partial t_{\mathcal{X}}} & 0 & 0 & 0 \\ \frac{\partial q_{\mathcal{X}}'}{\partial t_{\mathcal{X}}} & \frac{\partial q_{\mathcal{X}}'}{\partial q_{\mathcal{X}}} & 0 & 0 \\ \frac{\partial q_{\mathcal{L}}'}{\partial t_{\mathcal{X}}} & 0 & \frac{\partial q_{\mathcal{L}}'}{\partial q_{\mathcal{L}}} & 0 \\ \frac{\partial q_{\mathcal{R}}'}{\partial t_{\mathcal{X}}} & 0 & 0 & \frac{\partial q_{\mathcal{R}}'}{\partial q_{\mathcal{R}}} \end{bmatrix}. \tag{6}$$

As J is triangular, its determinant $|J|$ is equal to the product of diagonal elements:

$$\begin{aligned} \ln|J| =& \ln\{\frac{\partial t_{\mathcal{X}}'}{\partial t_{\mathcal{X}}} \times \frac{\partial q_{\mathcal{X}}'}{\partial q_{\mathcal{X}}} \times \frac{\partial q_{\mathcal{L}}'}{\partial q_{\mathcal{L}}} \times \frac{\partial q_{\mathcal{R}}'}{\partial q_{\mathcal{R}}}\} \\ =& \ln 1 + \ln\ D\hat{F}\Big(\frac{t_{\mathcal{P}} - t_{\mathcal{X}}}{t_{\mathcal{P}} - t_{\mathcal{X}}'} \times \hat{F^{-1}}(q_{\mathcal{X}})\Big) + \ln \frac{\partial}{\partial q_{\mathcal{X}}} \frac{t_{\mathcal{P}} - t_{\mathcal{X}}}{t_{\mathcal{P}} - t_{\mathcal{X}}'} \hat{F^{-1}}\Big(q_{\mathcal{X}}\Big) \\ &+ \ln\ D\hat{F}\Big(\frac{t_{\mathcal{X}} - t_{\mathcal{L}}}{t_{\mathcal{X}}' - t_{\mathcal{L}}} \times \hat{F^{-1}}(q_{\mathcal{L}})\Big) + \ln \frac{\partial}{\partial q_{\mathcal{L}}} \frac{t_{\mathcal{X}} - t_{\mathcal{L}}}{t_{\mathcal{X}}' - t_{\mathcal{L}}} \hat{F^{-1}}\Big(q_{\mathcal{L}}\Big) \\ &+ \ln\ D\hat{F}\Big(\frac{t_{\mathcal{X}} - t_{\mathcal{R}}}{t_{\mathcal{X}}' - t_{\mathcal{R}}} \times \hat{F^{-1}}(q_{\mathcal{R}})\Big) + \ln \frac{\partial}{\partial q_{\mathcal{R}}} \frac{t_{\mathcal{X}} - t_{\mathcal{R}}}{t_{\mathcal{X}}' - t_{\mathcal{R}}} \hat{F^{-1}}\Big(q_{\mathcal{R}}\Big) \\ =& \ln\ D\hat{F}\Big(\frac{t_{\mathcal{P}} - t_{\mathcal{X}}}{t_{\mathcal{P}} - t_{\mathcal{X}}'} \times \hat{F^{-1}}(q_{\mathcal{X}})\Big) + \ln\ D\hat{F^{-1}}\Big(q_{\mathcal{X}}\Big) + \ln \frac{t_{\mathcal{P}} - t_{\mathcal{X}}}{t_{\mathcal{P}} - t_{\mathcal{X}}'} \\ &+ \ln\ D\hat{F}\Big(\frac{t_{\mathcal{X}} - t_{\mathcal{L}}}{t_{\mathcal{X}}' - t_{\mathcal{L}}} \times \hat{F^{-1}}(q_{\mathcal{L}})\Big) + \ln\ D\hat{F^{-1}}\Big(q_{\mathcal{L}}\Big) + \ln \frac{t_{\mathcal{X}} - t_{\mathcal{L}}}{t_{\mathcal{X}}' - t_{\mathcal{L}}} \\ &+ \ln\ D\hat{F}\Big(\frac{t_{\mathcal{X}} - t_{\mathcal{R}}}{t_{\mathcal{X}}' - t_{\mathcal{R}}} \times \hat{F^{-1}}(q_{\mathcal{R}})\Big) + \ln\ D\hat{F^{-1}}\Big(q_{\mathcal{R}}\Big) + \ln \frac{t_{\mathcal{X}} - t_{\mathcal{R}}}{t_{\mathcal{X}}' - t_{\mathcal{R}}}. \end{aligned} \tag{7}$$

The derivatives $D\hat{F}$ and $D\hat{F^{-1}}$ are computed using numerical approximations for the first and last pieces, or as the gradient of the linear approximation for internal pieces. As its final step, the operator returns $\ln|J|$.

Simple Distance

While `ConstantDistance` proposes internal node heights, `SimpleDistance` operates on the root. Let $\mathcal{X}$ be the root node and let $\mathcal{L}$ and $\mathcal{R}$ be its two children.

Step 1. Propose a new height for $t_{\mathcal{X}}$:

$$t_{\mathcal{X}}' \leftarrow t_{\mathcal{X}} + s\Sigma. \tag{8}$$

Ensure that $\max\{t_{\mathcal{L}}, t_{\mathcal{R}}\} < t_{\mathcal{X}}'$, and if the constraint is broken then reject the proposal.

Step 2. Propose new rate quantiles for the two children $\mathcal{C} \in \{\mathcal{L}, \mathcal{R}\}$:

$$\begin{aligned} q_{\mathcal{C}}{}' \leftarrow & \hat{F}\Big(r_{\mathcal{C}}{}'\Big) \\ \leftarrow & \hat{F}\Big(\frac{t_{\mathcal{X}} - t_{\mathcal{C}}}{t_{\mathcal{X}}{}' - t_{\mathcal{C}}} \times r_{\mathcal{C}}\Big) \\ \leftarrow & \hat{F}\Big(\frac{t_{\mathcal{X}} - t_{\mathcal{C}}}{t_{\mathcal{X}}{}' - t_{\mathcal{C}}} \times \hat{F^{-1}}(q_{\mathcal{C}})\Big). \end{aligned} \tag{9}$$

These proposals ensure that the genetic distance between $\mathcal{X}$ and its children $\mathcal{C}$ remain constant after the operation by enforcing the constraint:

$$r_{\mathcal{C}}(t_{\mathcal{X}} - t_{\mathcal{C}}) = r_{\mathcal{C}}{}'(t_{\mathcal{X}}{}' - t_{\mathcal{C}}). \tag{10}$$

Ensure that $0 < q_{\mathcal{C}}{}' < 1$, and if the constraint is broken then reject the proposal.

Step 3. Finally, in order to calculate the Metropolis-Hastings-Green ratio, return the determinant of the Jacobian matrix:

$$\begin{aligned} J = & \begin{bmatrix} \frac{\partial t_{\mathcal{X}}{}'}{\partial t_{\mathcal{X}}} & \frac{\partial t_{\mathcal{X}}{}'}{\partial q_{\mathcal{L}}} & \frac{\partial t_{\mathcal{X}}{}'}{\partial q_{\mathcal{R}}} \\ \frac{\partial q_{\mathcal{L}}{}'}{\partial t_{\mathcal{X}}} & \frac{\partial q_{\mathcal{L}}{}'}{\partial q_{\mathcal{L}}} & \frac{\partial q_{\mathcal{L}}{}'}{\partial q_{\mathcal{R}}} \\ \frac{\partial q_{\mathcal{R}}{}'}{\partial t_{\mathcal{X}}} & \frac{\partial q_{\mathcal{R}}{}'}{\partial q_{\mathcal{L}}} & \frac{\partial q_{\mathcal{R}}{}'}{\partial q_{\mathcal{R}}} \end{bmatrix} \\ = & \begin{bmatrix} \frac{\partial t_{\mathcal{X}}{}'}{\partial t_{\mathcal{X}}} & 0 & 0 \\ \frac{\partial q_{\mathcal{L}}{}'}{\partial t_{\mathcal{X}}} & \frac{\partial q_{\mathcal{L}}{}'}{\partial q_{\mathcal{L}}} & 0 \\ \frac{\partial q_{\mathcal{R}}{}'}{\partial t_{\mathcal{X}}} & 0 & \frac{\partial q_{\mathcal{R}}{}'}{\partial q_{\mathcal{R}}} \end{bmatrix}. \end{aligned} \tag{11}$$

As J is triangular, its determinant $|J|$ is equal to the product of diagonal elements:

$$\begin{aligned} \ln|J| = & \ln\{\frac{\partial t_{\mathcal{X}}{}'}{\partial t_{\mathcal{X}}} \times \frac{\partial q_{\mathcal{L}}{}'}{\partial q_{\mathcal{L}}} \times \frac{\partial q_{\mathcal{R}}{}'}{\partial q_{\mathcal{R}}}\} \\ = & \ln\frac{\partial t_{\mathcal{X}}{}'}{\partial t_{\mathcal{X}}} + \ln\frac{\partial q_{\mathcal{L}}{}'}{\partial q_{\mathcal{L}}} + \ln\frac{\partial q_{\mathcal{R}}{}'}{\partial q_{\mathcal{R}}} \\ = & \ln 1 \\ & + \ln\ D\hat{F}\Big(\frac{t_{\mathcal{X}} - t_{\mathcal{L}}}{t_{\mathcal{X}}{}' - t_{\mathcal{L}}} \times \hat{F^{-1}}(q_{\mathcal{L}})\Big) + \ln\frac{\partial}{\partial q_{\mathcal{L}}}\frac{t_{\mathcal{X}} - t_{\mathcal{L}}}{t_{\mathcal{X}}{}' - t_{\mathcal{L}}}\hat{F^{-1}}\Big(q_{\mathcal{L}}\Big) \\ & + \ln\ D\hat{F}\Big(\frac{t_{\mathcal{X}} - t_{\mathcal{R}}}{t_{\mathcal{X}}{}' - t_{\mathcal{R}}} \times \hat{F^{-1}}(q_{\mathcal{R}})\Big) + \ln\frac{\partial}{\partial q_{\mathcal{R}}}\frac{t_{\mathcal{X}} - t_{\mathcal{R}}}{t_{\mathcal{X}}{}' - t_{\mathcal{R}}}\hat{F^{-1}}\Big(q_{\mathcal{R}}\Big) \\ = & \ln\ D\hat{F}\Big(\frac{t_{\mathcal{X}} - t_{\mathcal{L}}}{t_{\mathcal{X}}{}' - t_{\mathcal{L}}} \times \hat{F^{-1}}(q_{\mathcal{L}})\Big) + \ln\ D\hat{F^{-1}}\Big(q_{\mathcal{L}}\Big) + \ln\frac{t_{\mathcal{X}} - t_{\mathcal{L}}}{t_{\mathcal{X}}{}' - t_{\mathcal{L}}} \\ & + \ln\ D\hat{F}\Big(\frac{t_{\mathcal{X}} - t_{\mathcal{R}}}{t_{\mathcal{X}}{}' - t_{\mathcal{R}}} \times \hat{F^{-1}}(q_{\mathcal{R}})\Big) + \ln\ D\hat{F^{-1}}\Big(q_{\mathcal{R}}\Big) + \ln\frac{t_{\mathcal{X}} - t_{\mathcal{R}}}{t_{\mathcal{X}}{}' - t_{\mathcal{R}}}. \end{aligned} \tag{12}$$

As its final step, the operator returns $\ln|J|$.

Small Pulley

Just like the previous operator, `SmallPulley` operates on the root. Let $\mathcal{X}$ be the root node and let $\mathcal{L}$ and $\mathcal{R}$ be its two children. However, unlike `SimpleDistance`, this operator alters the two genetic distances $d_{\mathcal{L}} = r_{\mathcal{L}}(t_{\mathcal{X}} - t_{\mathcal{L}}) = \hat{F^{-1}}(q_{\mathcal{L}})(t_{\mathcal{X}} - t_{\mathcal{L}})$ and $d_{\mathcal{R}} = r_{\mathcal{R}}(t_{\mathcal{X}} - t_{\mathcal{R}}) = \hat{F^{-1}}(q_{\mathcal{R}})(t_{\mathcal{X}} - t_{\mathcal{R}})$, while conserving their sum $d_{\mathcal{L}} + d_{\mathcal{R}}$.

Step 1. Propose new genetic distances for $d_{\mathcal{L}}$ and $d_{\mathcal{R}}$:

$$d_{\mathcal{L}}' \leftarrow d_{\mathcal{L}} + s\Sigma \tag{13}$$

$$d_{\mathcal{R}}' \leftarrow d_{\mathcal{R}} - s\Sigma \tag{14}$$

Ensure that $0 < d_{\mathcal{L}}' < d_{\mathcal{L}} + d_{\mathcal{R}}$, and if the constraint is broken then reject the proposal.

Step 2. Propose new rate quantiles for the two children $\mathcal{L}$ and $\mathcal{R}$:

$$\begin{aligned} q_{\mathcal{L}}' &\leftarrow \hat{F}\Big(\frac{d_{\mathcal{L}}'}{t_{\mathcal{X}} - t_{\mathcal{L}}}\Big) \\ &\leftarrow \hat{F}\Big(\frac{\hat{F^{-1}}(q_{\mathcal{L}})(t_{\mathcal{X}} - t_{\mathcal{L}}) + \Sigma}{t_{\mathcal{X}} - t_{\mathcal{L}}}\Big) \end{aligned} \tag{15}$$

$$\begin{aligned} q_{\mathcal{R}}' &\leftarrow \hat{F}\Big(\frac{d_{\mathcal{R}}'}{t_{\mathcal{X}} - t_{\mathcal{R}}}\Big) \\ &\leftarrow \hat{F}\Big(\frac{\hat{F^{-1}}(q_{\mathcal{R}})(t_{\mathcal{X}} - t_{\mathcal{R}}) - \Sigma}{t_{\mathcal{X}} - t_{\mathcal{R}}}\Big). \end{aligned} \tag{16}$$

Step 3. Return the determinant of the Jacobian matrix:

$$\begin{aligned} J &= \begin{bmatrix} \frac{\partial q_{\mathcal{L}}'}{\partial q_{\mathcal{L}}} & \frac{\partial q_{\mathcal{L}}'}{\partial q_{\mathcal{R}}} \\ \frac{\partial q_{\mathcal{R}}'}{\partial q_{\mathcal{L}}} & \frac{\partial q_{\mathcal{R}}'}{\partial q_{\mathcal{R}}} \end{bmatrix} \\ &= \begin{bmatrix} \frac{\partial q_{\mathcal{L}}'}{\partial q_{\mathcal{L}}} & 0 \\ 0 & \frac{\partial q_{\mathcal{R}}'}{\partial q_{\mathcal{R}}} \end{bmatrix}. \end{aligned} \tag{17}$$

As J is triangular/diagonal, its determinant $|J|$ is equal to the product of diagonal elements:

$$
\begin{aligned}
\ln|J| &= \ln\{\frac{\partial q_{\mathcal{L}}'}{\partial q_{\mathcal{L}}} \times \frac{\partial q_{\mathcal{R}}'}{\partial q_{\mathcal{R}}}\} \\
&= \ln \frac{\partial q_{\mathcal{L}}'}{\partial q_{\mathcal{L}}} + \ln \frac{\partial q_{\mathcal{R}}'}{\partial q_{\mathcal{R}}} \\
&= \ln \ D\hat{F}\Big(\frac{F^{\hat{-1}}(q_{\mathcal{L}})(t_{\mathcal{X}} - t_{\mathcal{L}}) + \Sigma}{t_{\mathcal{X}} - t_{\mathcal{L}}}\Big) + \ln \frac{\partial q_{\mathcal{L}}'}{\partial q_{\mathcal{L}}} \frac{F^{\hat{-1}}(q_{\mathcal{L}})(t_{\mathcal{X}} - t_{\mathcal{L}}) + \Sigma}{t_{\mathcal{X}} - t_{\mathcal{L}}} \\
&\quad + \ln \ D\hat{F}\Big(\frac{F^{\hat{-1}}(q_{\mathcal{R}})(t_{\mathcal{X}} - t_{\mathcal{R}}) - \Sigma}{t_{\mathcal{X}} - t_{\mathcal{R}}}\Big) + \ln \frac{\partial q_{\mathcal{R}}'}{\partial q_{\mathcal{R}}} \frac{F^{\hat{-1}}(q_{\mathcal{R}})(t_{\mathcal{X}} - t_{\mathcal{R}}) - \Sigma}{t_{\mathcal{X}} - t_{\mathcal{R}}} \\
&= \ln \ D\hat{F}\Big(\frac{F^{\hat{-1}}(q_{\mathcal{L}})(t_{\mathcal{X}} - t_{\mathcal{L}}) + \Sigma}{t_{\mathcal{X}} - t_{\mathcal{L}}}\Big) + \ln \ DF^{\hat{-1}}(q_{\mathcal{L}}) \\
&\quad + \ln \ D\hat{F}\Big(\frac{F^{\hat{-1}}(q_{\mathcal{R}})(t_{\mathcal{X}} - t_{\mathcal{R}}) - \Sigma}{t_{\mathcal{X}} - t_{\mathcal{R}}}\Big) + \ln \ DF^{\hat{-1}}(q_{\mathcal{R}}). \qquad (18)
\end{aligned}
$$

Thus, as its final step, the operator returns $\ln|J|$.

3 CisScale operator

`CisScale` was originally introduced by Zhang and Drummond 2020 for the *real* parameterisation (therein named ucldstdevScaleOperator). Under the *quant* configuration, the `CisScale` operator works as follows.

Step 1. Propose a new value for the relaxed clock standard deviation σ

$$\sigma' \leftarrow \sigma \times e^{s\Sigma}. \tag{19}$$

Step 2. Recalculate all branch substitution rate quantiles q such that their rates r remain constant

$$\text{let } r = \hat{F}^{-1}(q|\sigma) \tag{20}$$

$$\text{let } r' = r \tag{21}$$

$$q' \leftarrow \hat{F}(r'|\sigma') = \hat{F}(\hat{F}^{-1}(q|\sigma)|\sigma'). \tag{22}$$

Step 3. Return the log Hastings-Green ratio of this proposal. If Σ was drawn from a symmetric proposal kernel (such as the Bactrian distribution), this is equal to:

$$|J| = \log(e^{s\Sigma}) + \log\left(\frac{\delta}{\delta q}\hat{F}(\hat{F}^{-1}(q|\sigma)|\sigma')\right) \tag{23}$$

$$= s\Sigma + \log D\hat{F}(\hat{F}^{-1}(q|\sigma)|\sigma') + \log D\hat{F}^{-1}(q|\sigma), \tag{24}$$

where derivatives $D\hat{F}$ and $D\hat{F}^{-1}$ can be approximated using either the piecewise linear model or standard numerical libraries.

4 Narrow exchange rates

The `NarrowExchangeRate` operator is also compatible with rate quantiles. This operator behaves the same as presented in the main article however the Hastings-Green ratio requires further augmentation due to changes in dimension throughout the proposal.

Step 1. Apply `NarrowExchange` to the current tree topology as described in the main article. This will return a Hastings ratio H due to the asymmetry of this proposal.

Step 2. Compute the relevant branch rates r_i for $r \in \{A, B, C, D\}$ of the current state from their respective quantile parameters.

$$r_i = \hat{F}^{-1}(q_i). \tag{25}$$

Step 3. Propose new rates and node heights and compute the Hastings-Green ratio of the real-space component of the proposal (e.g. Algorithms 1-2 of the main article).

$$(r'_A, r'_B, r'_C, r'_D, t'_D, |J_r|) \leftarrow \texttt{PROPOSAL}(r_A, r_B, r_C, r_D, t_D). \tag{26}$$

Step 4. Transform the rates back into quantiles.

$$q'_i = \hat{F}(r'_i). \tag{27}$$

Step 5. Compute the log Hastings-Green ratio of the interconversion between rates and quantiles.

$$\log |J_q| = \log \hat{F}(q) + \log \hat{F}^{-1}(r'). \tag{28}$$

Step 6. Return the total log Hastings-Green ratio of this proposal: $\log H + \log |J_r| + \log |J_q|$.

5 Summary of proposal kernels

Operators whose proposal kernels are affected by the decision to use a Bactrian kernel, as opposed to a uniform kernel, are specified below.

	Operator(s)	Proposal	Parameter x
1	`RandomWalk`	$x' \leftarrow x + s\Sigma$	$\vec{\mathcal{R}}, \sigma$
2	`Scale`	$x' \leftarrow x \times e^{s\Sigma}$	$\vec{\mathcal{R}}, \sigma$
3	`Interval`	$y \leftarrow \frac{1-x}{x} \times e^{s\Sigma}$ $x' \leftarrow \frac{y}{y+1}$	$\vec{\mathcal{R}}$
4	`ConstantDistance` `SimpleDistance`	$x' \leftarrow x + s\Sigma$	t
5	`SmallPulley`	$x' \leftarrow x + s\Sigma$	$\vec{\mathcal{R}}$
6	`CisScale`	$x' \leftarrow x \times e^{s\Sigma}$	σ

Table 1: Proposal kernels $q(x'|x)$ of clock model operators. In each operator, Σ is drawn from either a Bactrian(m) or uniform distribution. The scale size s is tunable. `ConstantDistance` and `SimpleDistance` propose tree heights t. The `Interval` operator applies to rate quantiles and respects its domain i.e. $0 < x, x' < 1$.

6 Supplementary NER Algorithm

A second NER algorithm is presented below. This operator was rejected by the screening protocol on simulated data.

Algorithm 1 The NER$\{\mathcal{D}_{BC}, \mathcal{D}_{CE}\}$ operator.

procedure PROPOSAL(t_A, t_B, t_C, t_D, t_E, r_A, r_B, r_C, r_D)

  $s\Sigma \leftarrow$ getRandomWalkSize() ▷ Random walk size is 0 unless this is NERw
  $t'_D \leftarrow t_D + s\Sigma$ ▷ Propose new node height for D

  $r'_A \leftarrow r_A$ ▷ Propose new rates
  $r'_B \leftarrow \frac{r_B(t_D - t_B) + r_D(t_E - t_D) + r_D(t_E - t'_D)}{t'_D - t_B}$
  $r'_C \leftarrow \frac{r_C(t_E - t_C) - r_D(t_E - t'_D)}{t'_D - t_C}$
  $r'_D \leftarrow r_D$

  $|J| \leftarrow \frac{(t_D - t_B)(t_E - t_C)}{(t'_D - t_B)(t'_D - t_C)}$ ▷ Calculate Jacobian determinant
  return $(r'_A, r'_B, r'_C, r'_D, t'_D, |J|)$

References

[1] Zhang R, Drummond A. Improving the performance of Bayesian phylogenetic inference under relaxed clock models. BMC Evolutionary Biology. 2020;20:1–28.
