# Supplementary material for: Adaptive dating and fast proposals: Revisiting the phylogenetic relaxed clock model
Source: PLoS Comput Biol. 2021 Feb 2;17(2):e1008322. doi: 10.1371/journal.pcbi.1008322 (PMC7880504; doi:10.1371/journal.pcbi.1008322)
Supplement: S2 Appendix — Methodologies are validated using well-calibrated simulation studies. (PDF) [file pcbi.1008322.s002.pdf]

Adaptive dating and fast proposals: revisiting the phylogenetic relaxed clock model

Jordan Douglas[1,2*], Rong Zhang[1,2], Remco Bouckaert[1,2,3]

1 Centre for Computational Evolution, University of Auckland, Auckland, New Zealand
2 School of Computer Science, University of Auckland, Auckland, New Zealand
3 Max Planck Institute for the Science of Human History, Jena, Germany

* jordan.douglas@auckland.ac.nz

S2 Appendix: Well-calibrated simulation studies

The methods presented and benchmarked in this paper were validated with well-calibrated simulation studies using the same model and operator scheme that was benchmarked. This was achieved using 100 simulated datasets (each with $N = 30$ taxa and a $L = 2000$ nt alignment). The 95% highest posterior density (HPD) intervals of each parameter during MCMC were calculated, and these intervals were compared with the 'true' parameter values which the data was simulated under.

The validation results are presented as plots (below). In each plot, blue and red vertical lines are 95% HPD intervals, where blue intervals contain the true value and red intervals do not. The number at the top of each plot is the coverage; that is the percentage of simulations where the 'true' parameter value is within the 95% HPD interval. If all parameters have coverage close to 95%, then this suggests correctness of both methodology and implementation.

Overall, these experiments suggest that the operators presented in the main paper were correctly implemented.

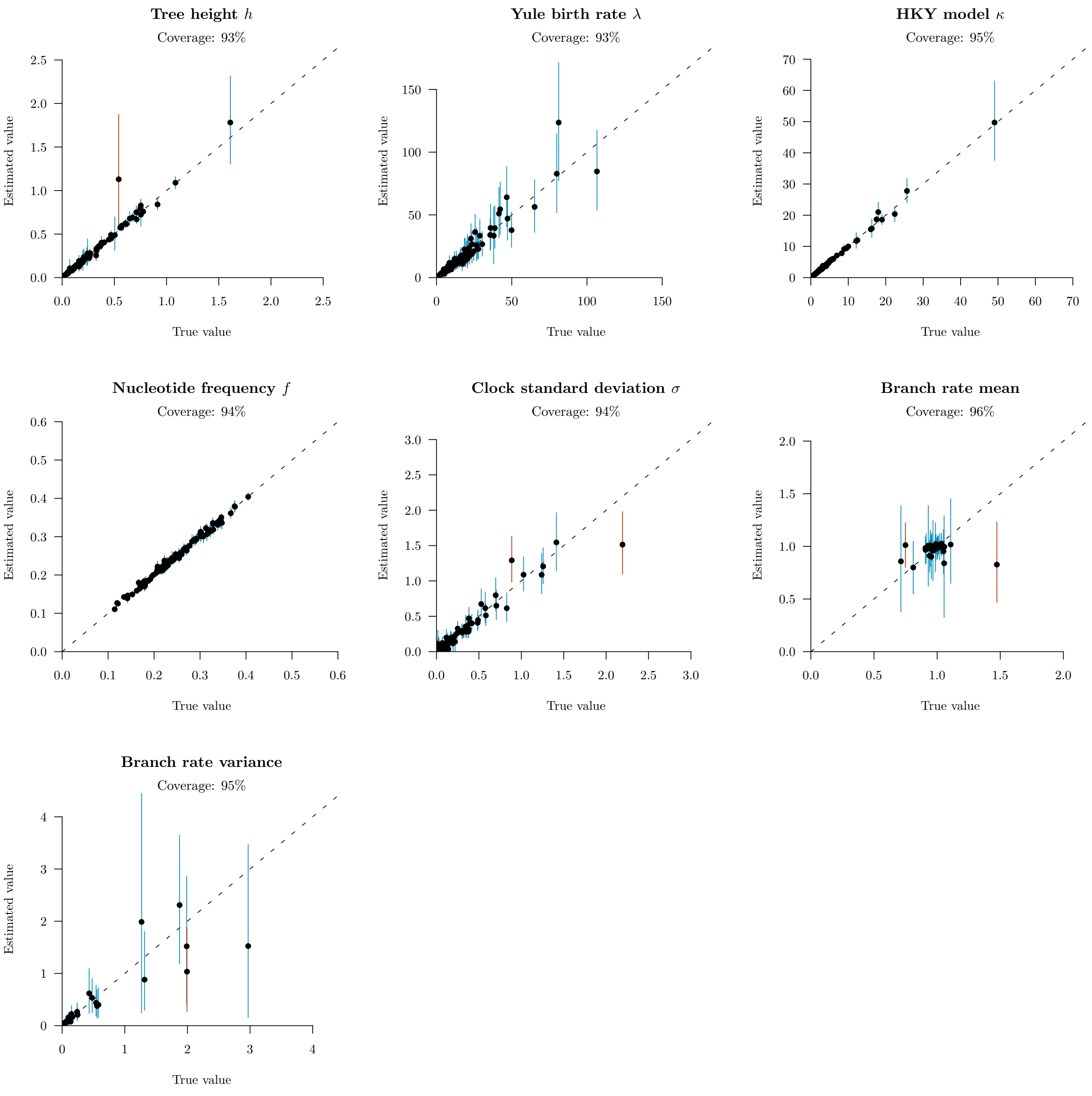

Figure 1: **The adapt (*cat*) configuration.**

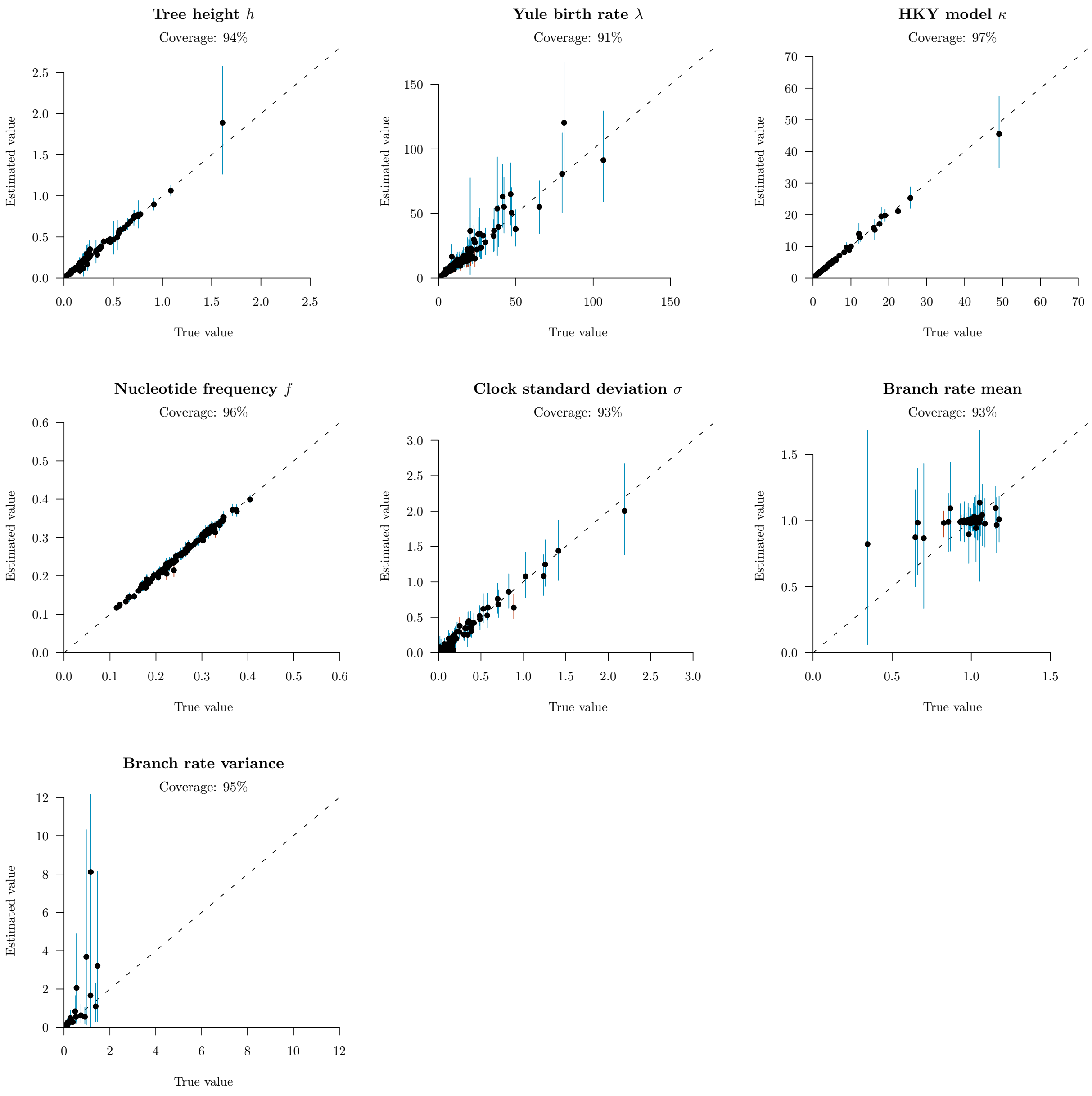

Figure 2: **The adapt (*real*) configuration.**

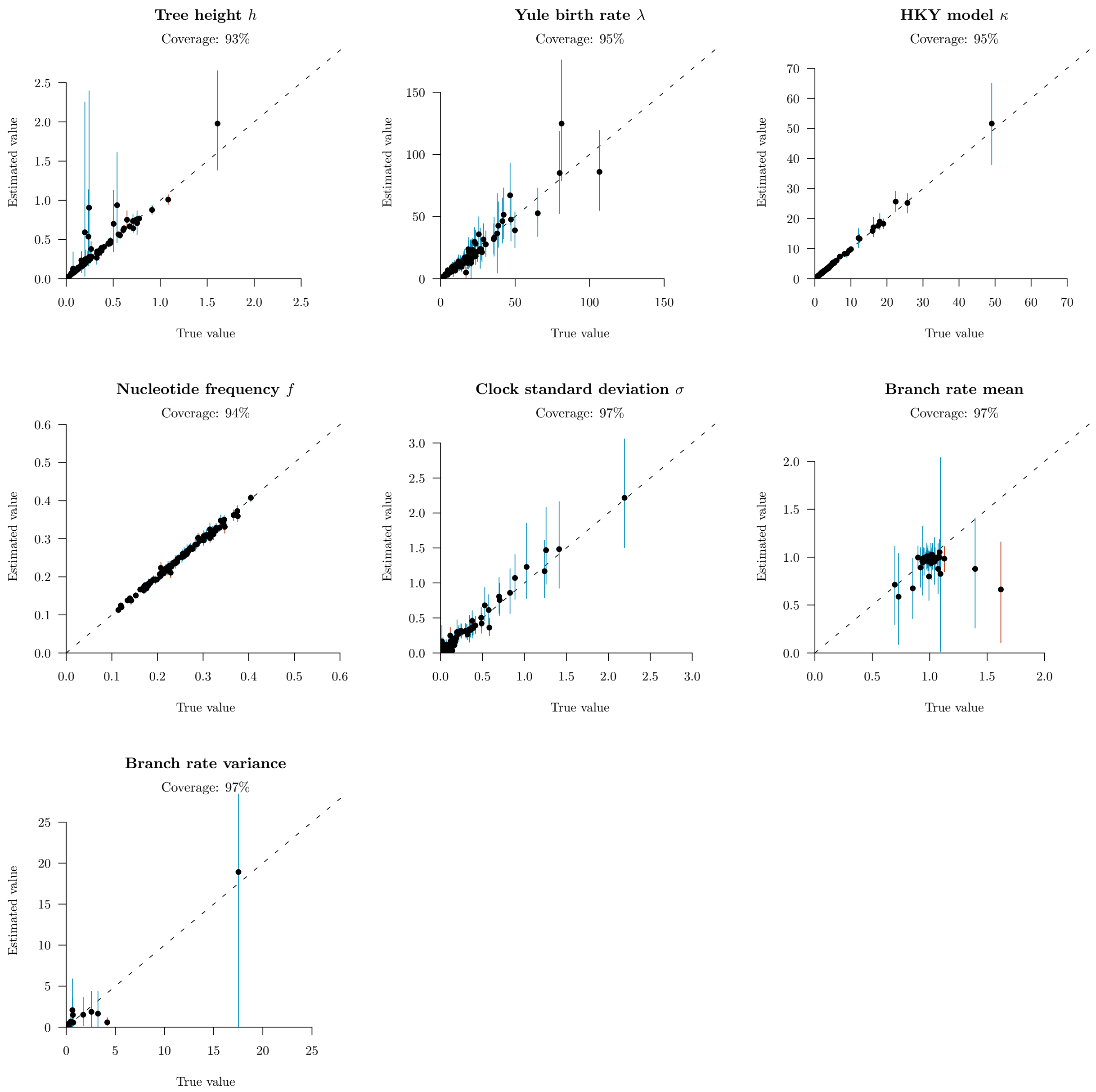

Figure 3: **The adapt (*quant*) configuration.** The constant distance operators were extended from *real* to *quant* as described in **S1 Appendix**.

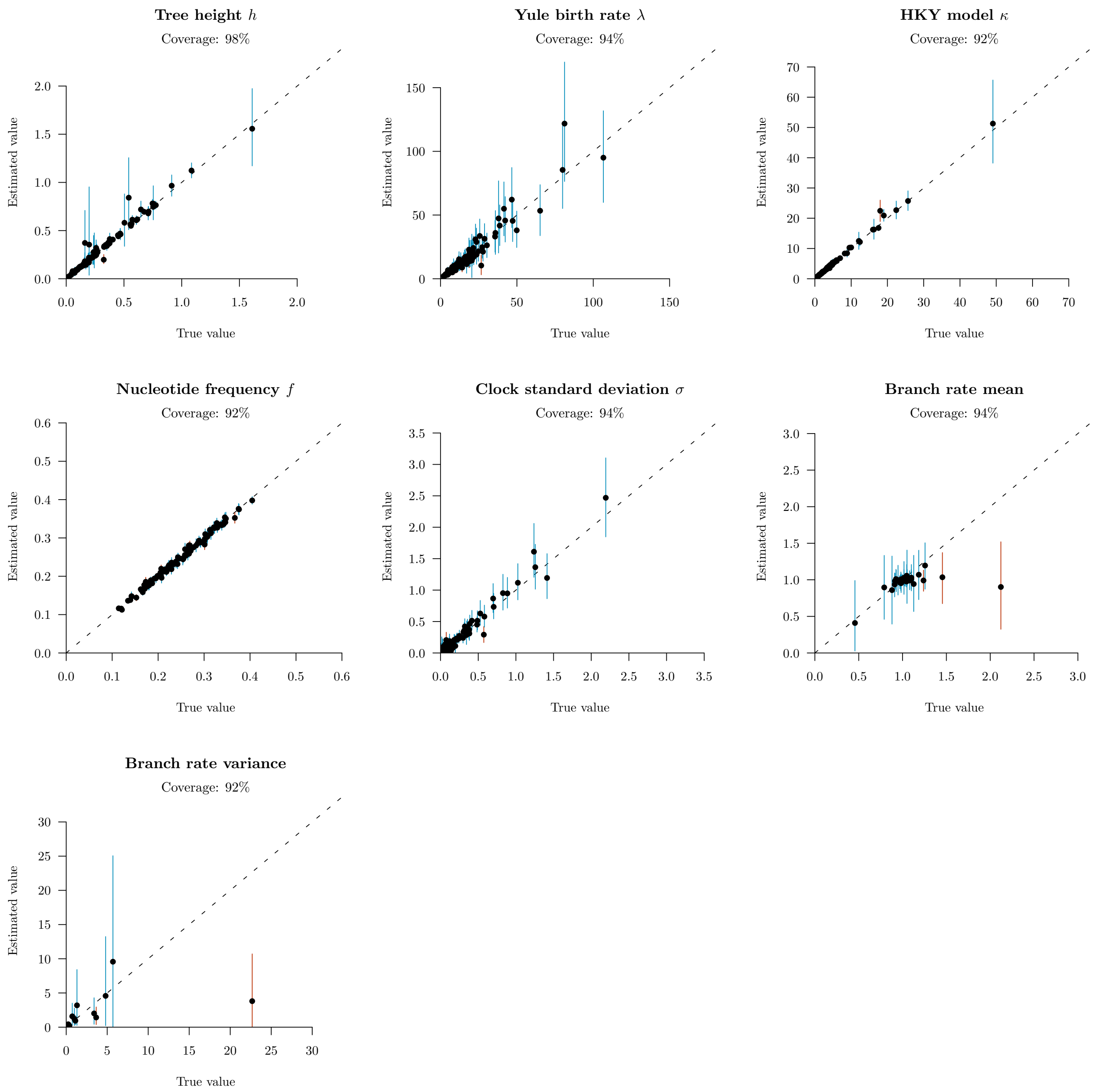

Figure 4: **The AVMVN configuration.** The adaptive NER operator, Bactrian proposals, and the *real* configuration are also included.
