# Supplementary figures and images for: Adaptive dating and fast proposals: Revisiting the phylogenetic relaxed clock model
Source: PLoS Comput Biol. 2021 Feb 2;17(2):e1008322. doi: 10.1371/journal.pcbi.1008322 (PMC7880504; doi:10.1371/journal.pcbi.1008322)

**cons(*quant*) vs. nocons(*quant*)**

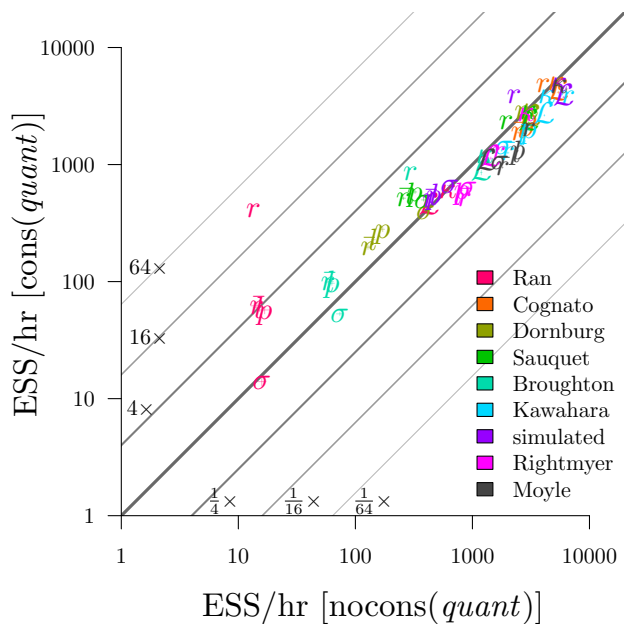

**adapt(*quant*) vs. nocons(*quant*)**

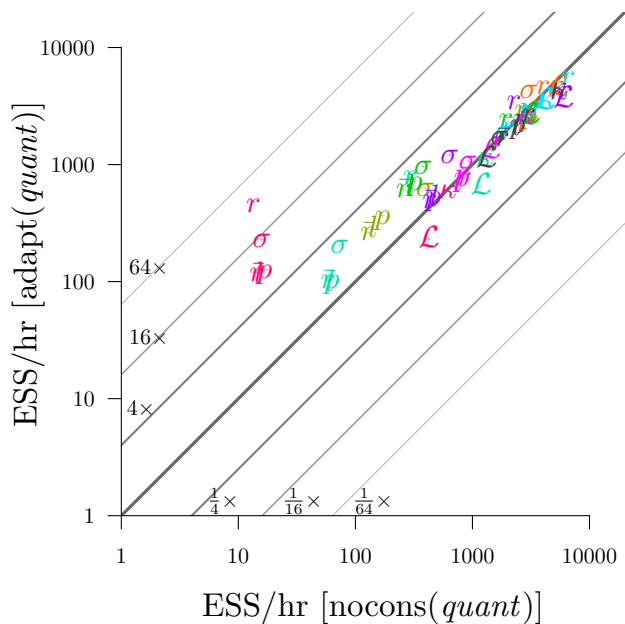

**adapt(*quant*) vs. cons(*quant*)**

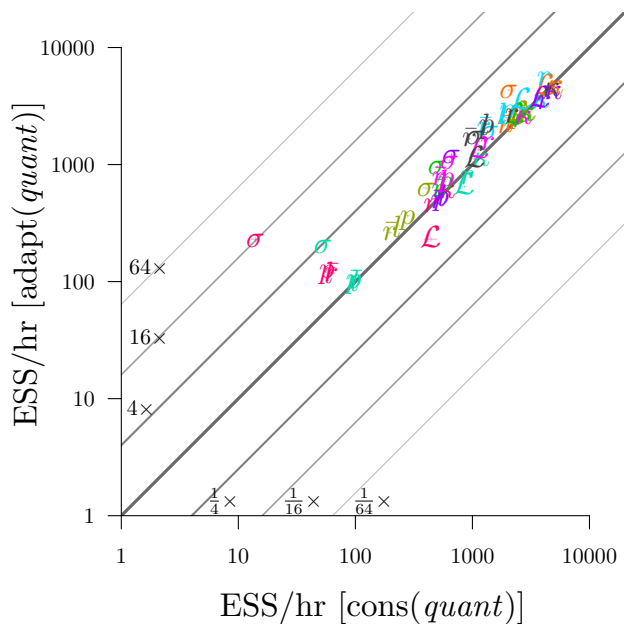

**adapt(*cat*) vs. *cat***

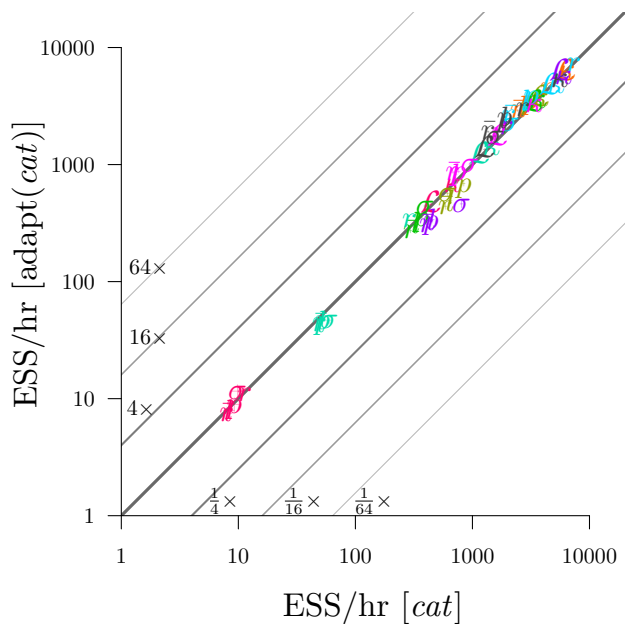

Supplement: S1 Fig — These results show that cat does not benefit from adaptive weight sampling. Whereas, adapt and cons both greatly improve the quant parameterisation for most datasets, as expected. (PDF) [file pcbi.1008322.s003.pdf]
