# Supplementary material for: Adaptive dating and fast proposals: Revisiting the phylogenetic relaxed clock model
Source: PLoS Comput Biol. 2021 Feb 2;17(2):e1008322. doi: 10.1371/journal.pcbi.1008322 (PMC7880504; doi:10.1371/journal.pcbi.1008322)
Supplement: Attachment — Submitted filename: reviewer_response.pdf [file pcbi.1008322.s005.pdf]

2 November 2020

To whom it may concern,

We wish to thank the reviewers for their helpful feedback, and the editors for considering our article titled "*Adaptive dating and fast proposals: revisiting the phylogenetic relaxed clock model*" for publication in PLOS Computational Biology. Please find attached our responses to points made by the reviewers. Our changes are highlighted on the supplementary manuscript.

1 Minor revisions

1. "*In the Abstract, I suggest adding a brief explanation of uncorrelated relaxed clocks (for readers unfamiliar with the term).*" **Response:** We have now included a brief explanation.

2. "*In the Author Summary, I suggest mentioning molecular dating or divergence time estimation, which is the main aim when using relaxed clock models.*" **Response:** We have included a brief mention.

3. "*In the first paragraph of the Introduction, it should be noted that the molecular clock is only essential to molecular dating, but not to phylogenetic analysis in general. Clock models are rarely used in phylogenetic analysis unless one of the goals is to infer the divergence times. BEAST is a notable exception because it always implements some form of clock model. In contrast, the clock models in MrBayes are quite rarely used.*" **Response:** We have clarified that we are referring to clock-model-based phylogenetics in the first paragraph.

4. "*In the fourth paragraph of the Introduction, it would be reasonable to cite the work of Thorne et al. (1998, Mol Biol Evol), who presented the first Bayesian relaxed (autocorrelated) clock and also described a likelihood approximation to reduce computational burden. One other consideration is whether autocorrelated or uncorrelated models are more biologically relevant. This has been considered in a number of studies, including by Lepage et al. (2007, cited by the authors).*" **Response:** Thank you for your recommendations. These articles have been incorporated into the introduction.

5. "*In the first paragraph of the Methods, presumably theta represents the parameters of the branching or coalescent model used to generate the tree prior. It would be useful to clarify this.*" **Response:** Thank you for pointing this out. We have clarified this.

6. "*In the first paragraph of the section Clock Model Operators, please include an explanation of how the parameter is learned over the course of the MCMC. Is this tuned to achieve a target acceptance rate?.*" **Response:** This is now specified.

7. "*Given the superior performance of the real parameterisation for the rate, should this be adopted as the default in BEAST rather than cat?*" **Response:** We have added a new section at the end of the Discussion to cover this point as well as the next point below.

8. "*In the Discussion it would be helpful for the authors to describe some potential avenues for improving the proposed operator configuration .*" **Response:** See above.

9. "*Data points are not included on github.*" **Response:** Benchmarking results are now linked to at the bottom of the GitHub readme file (https://github.com/jordandouglas/ORC).

10. "*It is not fully clear why the optimization protocol follows the order as summarized in Figure 7, for example why the adaptive operator sampling precedes substitution rate parameterization – it is not clear that the former is more "fundamental" than the latter, and the sequence would affect the entire optimization results.*" **Response:** Rounds 1 and 2 (adapt and parameterisation) are really just 1 big round because all combinations of tests were performed. Therefore the ordering between the two doesn't matter in this case. This has been clarified at the beginning of the 'Round 2...' section. The selection of a rate parameterisation is very fundamental to the analysis because unlike the use of uniform/bactrian kernels (Round 3) or the use/no use of advanced operators (Rounds 4 and 5), there is not really a baseline or default to use until in the interim until reaching the round where it is benchmarked. Furthermore, the operators discussed in Rounds 4 and 5 are not compatible with the categories configuration. This has been clarified at the end of the "Round 2" section.

11. "*Page 2: You mention the strict clock, relaxed correlated and uncorrelated clock models. But you're not saying anything about fixed local clock model.*" **Response:** We have incorporated this into paragraph 4 of the introduction.

12. "*Page 23, Line 67: The sentence '$q(a|b)$: is the transition kernel' It would make more sense to write $q(x|x')$ to follow the same notation from the paragraph.*" **Response:** This has now been corrected.

13. "*Fig 1: Why is the prior density function not written as $p(R|)$?*" **Response:** Capital-r ($\mathcal{R}$) is an abstracted rate while little-r (r) is a function for transforming $\mathcal{R}$ into an unabstracted rate, and is also used as shorthand notation to mean the rate itself i.e. $r = r(\mathcal{R})$. This has been made clearer in the x-axis of this figure.

14. "*In general, for the tables. Tables require a label and brief descriptive title to be placed above the table and other texts, such as the legend and footnotes below the table.*" **Response:** Thank you for pointing this out, we have restructured all of the tables.

15. "*Table 1: Why not using t as abbreviation for node height as in Table 2?*" **Response:** Thank you for pointing out this inconsistency. It has been corrected.

16. "*Page 23, Line 336: would it be better to use a term other than 'explosion'?*" **Response:** This has now been rephrased.

17. "*Page 27, Paragraph 'Round 2...': Would it be possible that the observed effect is inverted by using a too large L? .*" **Response:** While we have not tested on extremely large L, this is not consistent with our findings that show a reasonably strong relationship between L and convergence rate (e.g. see Fig 9 bottom right panel). Our claim that "constant distance operators would likely be even stronger for larger L" also has a theoretical basis because likelihood functions become increasingly peaked with larger L (Fig 2).

2 Length of the article

Reviewer 1 has suggested that the article has "*An excessive number of figures and tables, and the level of detail in the Methods and Results can be cut down*". They have recommended areas where the article can be trimmed such as by combining Tables listing operators and removing Figure 13 as it is non-essential.

On the other hand, Reviewer 2 suggested that "*The inclusion of appropriate figures and tables help the reader to follow the relatively dense material covered in this manuscript (especially Tables 6, 7 and Fig. 7)*".

Overall, we have trimmed down the article in the following areas:

1. We have moved Algorithm 1 to Appendix S1 as this was not necessary for the article.
2. We have also moved Table 3 to Appendix S1 (this table details how proposal kernels are applied to various operators).
3. We have combined Tables 2, 4, and 5 into one table (now referred to as Table 2).
4. We have combined Fig 12 and 13 into one figure as they are part of the same round. We believe that showing a real phylogenetic tree inferred from empirical data (i.e. Fig 13), although mentioned only briefly in the main text, serves as an example which may help some readers to better understand the model without having to go through equations. Therefore we would prefer to keep this figure in the main article. It is also useful to understand *why* an operator does well and this is the key point of Fig 13.

Thank you for your consideration.
J. Douglas, R. Zhang, R. Bouckaert
