# Supplementary material for: Adaptive dating and fast proposals: Revisiting the phylogenetic relaxed clock model
Source: PLoS Comput Biol. 2021 Feb 2;17(2):e1008322. doi: 10.1371/journal.pcbi.1008322 (PMC7880504; doi:10.1371/journal.pcbi.1008322)
Supplement: Attachment — Submitted filename: Review Relaxed Clock Model.docx [file pcbi.1008322.s004.docx]

**Review -** Adaptive dating and fast proposal: revisiting the phylogenetic relaxed clock model

The authors did a very thorough job introducing all the relaxed clock model operators used in analyzing whether an adaptive operator can lead to an improved estimation of molecular substitution rates using the uncorrelated relaxed clock model. The inclusion of appropriate figures and tables help the reader to follow the relatively dense material covered in this manuscript (especially Tables 6,7 and Fig. 7). Certain touches like the “Round” headings helped to focus and summarize the workflow covered by the manuscript.

Major point:

1. It is not fully clear why the optimization protocol follows the order as summarized in Figure 7, for example why the adaptive operator sampling precedes substitution rate parameterization—it is not clear that the former is more “fundamental” than the latter, and the sequence would affect the entire optimization results.

Minor points:

1. Page 2: You mention the strict clock, relaxed correlated and uncorrelated clock models. But you’re not saying anything about fixed local clock model.
2. Page 23, Line 67: The sentence “q(a|b): is the transition kernel” It would make more sense to write q(x|x’) to follow the same notation from the paragraph.
3. Fig 1: Why is the prior density function not written as p(R|$\mathcal{o}$)?
4. In general, for the tables. Tables require a label and brief descriptive title to be placed above the table and other texts, such as the legend and footnotes below the table.
5. Table 1: Why not using t as abbreviation for node height as in Table 2?
6. Page 23, Line 336: would it be better to use a term other than “explosion”?
7. Page 27, Paragraph “Round 2…”: Would it be possible that the observed effect is inverted by using a too large L?
